# Use of US Public Health Travel Restrictions during COVID-19 Outbreak on Diamond Princess Ship, Japan, February–April 2020

**DOI:** 10.3201/eid2703.203820

**Published:** 2021-03

**Authors:** Alexandra M. Medley, Barbara J. Marston, Mitsuru Toda, Miwako Kobayashi, Michelle Weinberg, Leah F. Moriarty, M. Robynne Jungerman, Amethyst Clare A. Surpris, Barbara Knust, Anna M. Acosta, Caitlin E. Shockey, David Daigle, Zachary D. Schneider, Julia Charles, Atsuyoshi Ishizumi, Andrea Stewart, Laura A Vonnahme, Clive Brown, Stefanie White, Nicole J. Cohen, Marty Cetron

**Affiliations:** Centers for Disease Control and Prevention, Atlanta, Georgia, USA

**Keywords:** COVID-19, SARS-CoV-2, severe acute respiratory syndrome coronavirus 2, viruses, respiratory infections, zoonoses, coronavirus disease, United States, pandemic, epidemic, public health, travel restrictions, cruise ship, cluster, Diamond Princess, Japan

## Abstract

Public health travel restrictions (PHTR) are crucial measures during communicable disease outbreaks to prevent transmission during commercial airline travel and mitigate cross-border importation and spread. We evaluated PHTR implementation for US citizens on the Diamond Princess during its coronavirus disease (COVID-19) outbreak in Japan in February 2020 to explore how PHTR reduced importation of COVID-19 to the United States during the early phase of disease containment. Using PHTR required substantial collaboration among the US Centers for Disease Control and Prevention, other US government agencies, the cruise line, and public health authorities in Japan. Original US PHTR removal criteria were modified to reflect international testing protocols and enable removal of PHTR for persons who recovered from illness. The impact of PHTR on epidemic trajectory depends on the risk for transmission during travel and geographic spread of disease. Lessons learned from the Diamond Princess outbreak provide critical information for future PHTR use.

Public health travel restrictions (PHTR) have been used by the United States to reduce the likelihood of transmission of selected communicable diseases aboard aircraft (*1*). US federal mechanisms used to implement PHTR include the public health Do Not Board (DNB) list and the Public Health Border Lookout (PHLO) record (*2*,*3*). The DNB, established in 2007, prevents travelers who are contagious or potentially contagious with a communicable disease of public health concern from obtaining a boarding pass for any commercial flight within, to, or from the United States (*2*–*4*). The PHLO alerts Customs and Border Protection officials to notify the Centers for Disease Control and Prevention (CDC) when a person on PHTR attempts to enter the United States by any port of entry so public health action can be taken, if needed (*2*). The public health aspects are managed by CDC and implemented under the legal authority of the Department of Homeland Security (DHS) (*2*). State or local health departments, other federal agencies, or international partners may initiate PHTR requests by contacting CDC (*4*).

Certain criteria must be met before implementing PHTR (*2*). Primarily, the person must be known or believed to be infectious with, or at risk for becoming infectious with, a serious communicable disease that poses a public health threat to others during travel. If not, then >1 of the additional criteria must be met: the person is unaware of his or her diagnosis and cannot be notified by public health authorities, is not following public health recommendations, cannot be located, or is likely to travel on a commercial flight or travel internationally by any means; or PHTR are needed to respond to a public health outbreak or to help enforce a public health order. PHTR are removed when the person is no longer considered infectious or at risk for becoming infectious (*2*).

The outbreak of severe acute respiratory syndrome coronavirus 2 (SARS-CoV-2) infection on the Diamond Princess cruise ship in Japan in February 2020 was the earliest large-scale use of US PHTR applied to a cohort on the basis of a common exposure. A total of 111 individual PHTR were placed in 1 day, compared with 556 during the 10-year period 2007–2016 (*1*,*3*). Furthermore, placement of the largest single cohort on US federal PHTR previously comprised 14 persons identified as having had a high-risk exposure to Ebola virus during December 2014–April 2015 (*1*).

PHTR generally apply to both US citizens and foreign nationals and can be applied to persons located within the United States or abroad (*2*). CDC decided to limit use of PHTR to US citizens and residents on the Diamond Princess on the assumption that these persons had reason to return to the United States. In addition, the DHS implemented and managed travel restrictions for non–US citizens on the Diamond Princess, which are outside of the scope of this article. The use of PHTR for US citizens and residents on the Diamond Princess should also be differentiated from travel restrictions imposed by the US government by presidential proclamation under section 212(f) of the Immigration and Nationality Act that apply to certain immigrants or nonimmigrants (*5*).

## Coronavirus Disease Outbreak on the Diamond Princess

On February 3, 2020, the Diamond Princess cruise ship arrived in Yokohama, Japan, carrying 2,666 passengers and 1,045 crew (*6*). Two days earlier, 1 symptomatic passenger who departed the ship in Hong Kong had tested positive for severe acute respiratory syndrome coronavirus 2 (SARS-CoV-2), the causative agent of coronavirus disease (COVID-19). By February 5, additional passengers on the cruise ship tested positive for the virus; Japanese authorities instituted a 14-day onboard quarantine for all passengers. Effective quarantine of the crew was challenged by communal living quarters, few single-occupancy rooms for isolation, and the need for crew to continue performing essential duties (*7*). Passengers and crew testing positive for SARS-CoV-2 were transferred to hospital isolation wards, along with some of their family members who had not been tested or had tested negative. During Japan’s 14-day quarantine, public health authorities relocated passengers >80 years of age or with underlying conditions, along with passengers residing in windowless cabins, to land-based quarantine facilities (*7*).

Preliminary data suggested that although most transmission occurred before quarantine implementation, there was residual risk for transmission among crew members and among passengers sharing cabins (*8*,*9*). By February 18, Japan reported 531 confirmed cases (65 crew, 466 passengers) on the Diamond Princess, representing 14% of those on board; additional test results were pending. Concurrently, positive tests among passengers were declining, but positive cases were increasing among the crew (*7*). The overall infection rate on the Diamond Princess (19.2% of passengers and crew) exceeded the reported infection rate (110/100,000 population) in Hubei Province, and viral exposure risk was considered high for Diamond Princess passengers and crew (*6*;*10*–*14*). At that time, the United States had reported 15 confirmed cases of COVID-19 in 7 states, all imported or travel related, and travelers from Hubei Province were subject to mandatory quarantine (*15*). CDC decided that US citizens and residents on the Diamond Princess should not travel to the United States by commercial carrier until those testing positive for SARS-CoV-2 were no longer infectious and those never testing positive were no longer at risk of becoming infectious (*16*). 

In light of the apparent continuing spread of COVID-19 aboard the ship during the quarantine, and to prioritize US citizen welfare and safety, the US government offered a large-scale voluntary repatriation involving controlled movement of US citizens, permanent residents, and their partners from the Diamond Princess to the United States and encouraged all eligible to participate. A total of 329 persons disembarked the ship on February 16, evacuating by 2 chartered aircraft configured to prevent and control transmission on board. Except for 1 individual who recovered from COVID-19 in Japan, persons were placed in federally supervised mandatory quarantine by the US government for 14 days after arrival in the United States (*6*,*17*).

## PHTR for US Citizens from Diamond Princess in Japan

To prevent commercial travel to the United States by infectious persons, CDC placed all US citizens and residents remaining in Japan on PHTR. Before the repatriation, the US Embassy Tokyo, Mission Japan (USEMB Japan), informed US citizens and residents that those electing to remain in Japan would be placed on PHTR (*18*). CDC rapidly established an active monitoring process to confirm that persons not testing positive for SARS-CoV-2 remained asymptomatic and to enable PHTR removal for each person as soon as CDC’s criteria were met. More than 100 US citizens and residents remained in Japan. More than half were hospitalized for medical care due to SARS-CoV-2 infection or underlying health problems; others were spouses or travel companions of hospitalized patients, crew members, or persons who declined repatriation. Operational challenges of PHTR implementation for large cohorts during an epidemic have not been described. To evaluate the use of PHTR in this context and inform future use, we describe PHTR implementation during the Diamond Princess COVID-19 outbreak, including successes, challenges, and lessons learned.

## Methods

### Implementation and Removal of PHTR

We reconciled manifests from the US repatriation flights and the cruise ship and worked with USEMB Japan to identify and locate US citizens remaining in Japan, whether in hospitals, government quarantine facilities, or hotels; aboard the ship; or as residents of Japan. On February 19, the ship quarantine imposed by Japan’s authorities ended. All identified US citizens and legal permanent residents who had been on the Diamond Princess but declined repatriation were placed on PHTR; they were notified of the PHTR through the USEMB Japan and cruise ship by both email and letter.

**Figure 1:**
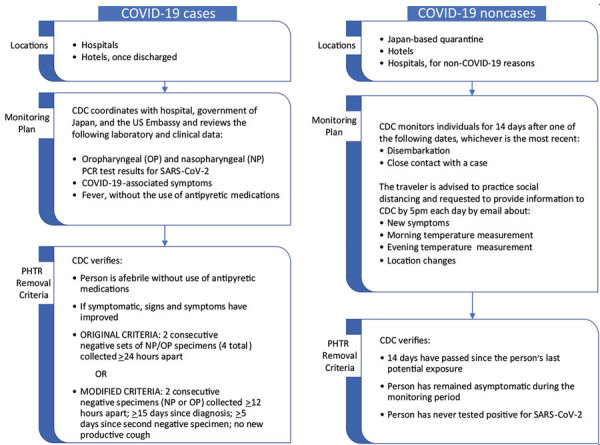
Implementation of PHTR for Diamond Princess cruise ship passengers and crew during the coronavirus disease outbreak, Japan, February 2020, including locations, monitoring plans, and criteria for PHTR removal for persons who tested positive for the virus (cases) and those who did not (non-cases). CDC, US Centers for Disease Control and Prevention; COVID-19, coronavirus disease; PHTR, public health travel restrictions; SARS-COV-2, severe acute respiratory syndrome coronavirus 2.

For passengers and crew who had not tested positive for SARS-CoV-2, CDC determined that commercial travel would be allowed after they had been off the ship for 14 days, provided they remained asymptomatic and had not tested positive for SARS-CoV-2 in the interim. To ensure these criteria were met, CDC implemented an active monitoring system until 14 days after the last potential exposure (i.e., disembarkation or close contact) (Figure 1; Appendix Figure 1, panel A). For those who experienced symptoms during the monitoring period, CDC and USEMB Japan facilitated medical evaluation in coordination with authorities in Japan.

For persons who tested positive for SARS-CoV-2, criteria to remove PHTR and discontinue isolation were based on CDC criteria in effect at the time: documentation of negative results in 2 consecutive sets of both oropharyngeal (OP) and nasopharyngeal (NP) specimens collected >24 hours apart; absence of fever without the use of antipyretic medications; and improvement in other symptoms (Figure 1; Appendix Figure 1, panel B). Hospital discharge criteria in use in Japan at the time required either 2 NP or 2 OP specimens collected >12 hours apart. For some patients, CDC was able to request the additional testing and sampling timeframe needed to meet CDC’s criteria. However, testing for SARS-CoV-2 in Japan at the time was covered under public funding and required approval of local public health centers (*19*,*20*). Obtaining additional tests to meet CDC criteria (criteria 1; Appendix Figure 1) was challenging for many hospitals, and CDC adopted modified criteria (criteria 2) on February 27 that accepted Japan’s testing strategy but added as criteria the times since first positive test (>15 days) and second negative test result (>5 days) and absence of a productive cough (Figure 1; Appendix Figure 1, panel C). Time-based criteria were determined from available CDC data that suggested viable virus was rarely recovered from patients later in their clinical course (*21*).

CDC coordinated with USEMB Japan and the cruise line to provide passengers, crew, and hospitals with the criteria and procedures for removal of PHTR (Appendix Figure 1). USEMB Japan consular staff monitored clinical status of all hospitalized US citizens, coordinating directly with hospitals and patients, and provided daily updates to CDC response team, which verified when patients met the criteria for discontinuation of PHTR and the anticipated date of removal. Japanese health workers, in close communication with USEMB Japan, assisted CDC active monitoring efforts for 9 passengers hospitalized for non–COVID-19 health concerns.

### Evaluation of PHTR

Using a database consolidating data from the cruise ship, the government of Japan, USEMB Japan, and CDC that was originally used for PHTR implementation, we described characteristics of persons subject to PHTR: US state of residence; sex; age; crew or passenger; history of close contact with a case (e.g., infected cabin mate); number of cabin mates; initial SARS-CoV-2 test result; presence of symptoms at the time of testing (symptomatic or asymptomatic), and disposition (entered monitoring or hospitalized). For those with COVID-19, we described their clinical severity and types of samples collected to meet the discharge criteria (OP, NP, or both), comparing proportions of those meeting original CDC criteria for PHTR removal to the modified criteria. We described outcomes, successes, and challenges of the monitoring process to identify lessons learned, based on our professional judgment and expertise on containing infectious diseases. We analyzed data using R (R Foundation for Statistical Computing, https://www.r-project.org). 

## Results

### Demographics and Dispositions of Persons with PHTR

CDC initially placed 108 US citizen passengers and crew on PHTR on February 19, 2020. One additional passenger self-declared as a legal permanent resident of the United States, and USEMB Japan identified 2 additional persons with dual citizenship, for a total of 111 PHTR. Thirteen persons did not live in the United States but reported secondary residences in or frequent travel to the United States. Of the 98 persons residing in the United States, 30 (31%) resided in California.

From the 111 US citizens and residents remaining in Japan, 44 entered active monitoring after disembarking the ship. Three of these persons were tested for SARS-CoV-2 during the active monitoring period, 1 because of a fever, 1 because of hospitalization for other reasons, and 1 for close contact to a confirmed case; 2 tested positive for SARS-CoV-2. Overall, 69 US citizens had positive test results for SARS-CoV-2 and were hospitalized in 35 hospitals in Japan (Table). Of these patients, 39 (57%) were symptomatic at the time of testing; some asymptomatic but ultimately testing positive US citizens disembarked the ship with test results still pending. Fourteen percent of all persons with COVID-19 were critically ill at any timepoint, including 1 patient with COVID-19 who became critically ill unrelated to COVID-19. The median age of COVID-19 patients was 71 years (range 25–92 years); median age of those testing negative for SARS-CoV-2 was 63 years (range 3–85 years; p = 0.005).

**Table:** Characteristics of Diamond Princess passengers and crew who remained in Japan after US repatriation with public health travel restrictions, 2020*

Characteristic	SARS-CoV-2 positive, no. (%), n = 69	SARS-CoV-2 negative, no. (%), n = 33	Odds ratio (95% CI)	p value
Sex				
F	38 (55)	18 (55)	Referent	None
M	31 (45)	15 (45)	1.02 (0.44–2.37)	0.96
Median age, y (range)	71 (25–92)	63 (3–85)		0.005†
Crew or passenger				
Passenger	66 (96)	33 (100)	ND	ND
Crew	3 (4)	0 (0)	ND	ND
Close contact				
Index patient in cabin	42 (63)	ND	ND	ND
Close contact	25 (37)	9 (100)	ND	ND
Room occupancy				
1	2 (3)	1 (3)	Referent	None
2	58 (88)	23 (70)	0.75 (0.06–24.36)	0.84
3	4 (6)	5 (15)	2.22 (0.13–87.42)	0.59
4	2 (3)	4 (12)	3.28 (0.17–144.84)	0.45
Disposition				
Entered monitoring	0	33 (100)	ND	ND
Criteria 1	40 (58)	0 (0)	ND	ND
Criteria 2	29 (42)	0 (0)	ND	ND

*Data were compiled for 111 US citizens and residents through April 7, 2020. The following characteristics were missing values: sex (2), age (3), room occupancy (11), SARS-CoV-2 test result (9). ND, no data; SARS-COV-2, severe acute respiratory syndrome coronavirus 2.  †By Wilcoxon rank-sum test.

### Removal of the PHTR

**Figure 2:**
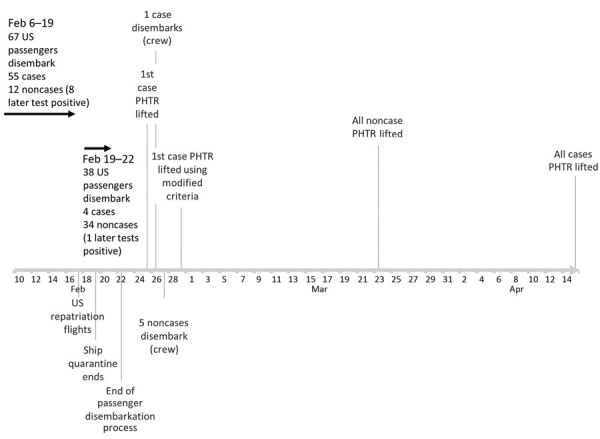
Significant disembarkation and monitoring events during a period of PHTR implemented for nonrepatriated US citizens and residents on the Diamond Princess cruise ship during the coronavirus disease pandemic, Japan, February 19–April 15, 2020. PHTR, public health travel restrictions.

**Figure 3:**
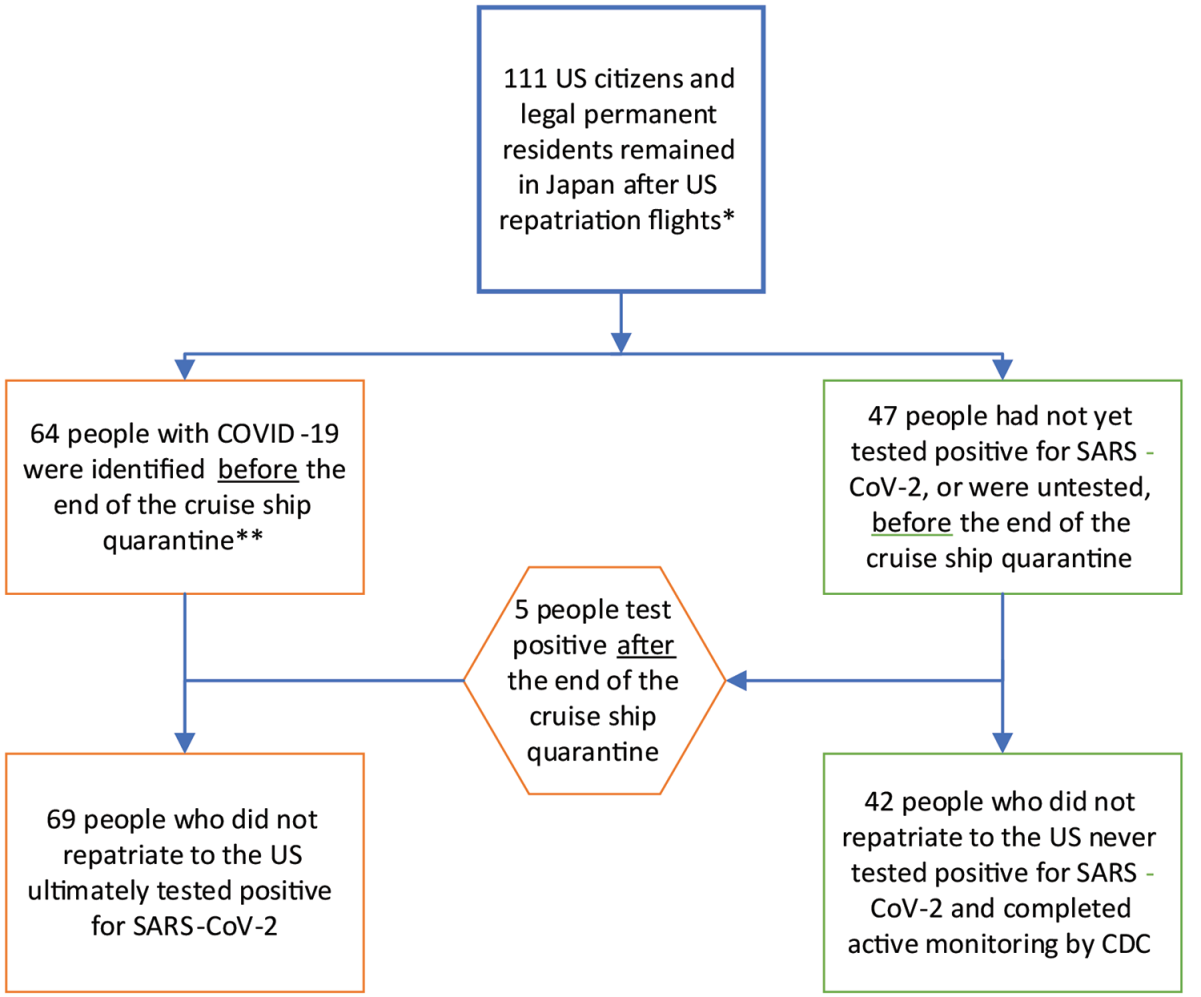
Final disposition (hospitalized for COVID-19 vs. entered active monitoring) of US citizen passengers and crew of the Diamond Princess cruise ship who remained in Japan following US repatriation flights and were subject to public health travel restrictions during the COVID-19 outbreak, 2020. Repatriation flights occurred on February 17, 2020. The Diamond Princess cruise ship quarantine mandated by Japan ended on February 19,^,^2020; by that date, 67 persons had disembarked, 3 of whom had not tested positive for SARS-CoV-2 at that time. CDC, US Centers for Disease Control and Prevention; COVID-19, coronavirus disease; SARS-COV-2, severe acute respiratory syndrome coronavirus 2.

US citizen and resident passengers disembarked February 19–22 and crew on February 27 (Figure 2). Of the 42 persons (37 passengers, 5 crew) never testing positive for SARS-CoV-2 (Figure 3), 40 (95.2%) completed active monitoring until 14 days after their last potential exposure, spending a median of 16 days with PHTR in place (range 6–34) depending on their disembarkation date (Appendix Figure 2). Two persons were unreachable, and CDC removed their PHTR 28 days (2 incubation periods) after the date of disembarkation.

**Figure 4:**
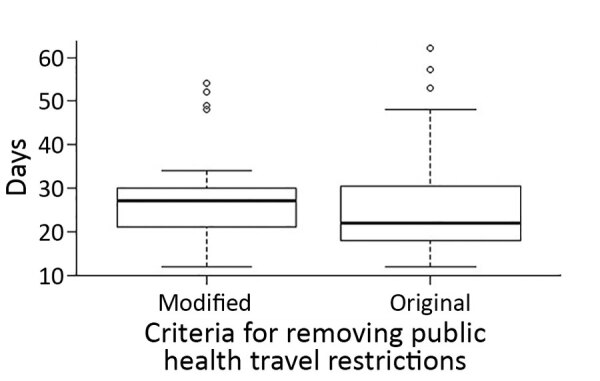
Whisker plot for days between notification of a positive SARS-CoV-2 test result and removal of PHTR for passengers and crew on board the Diamond Princess cruise ship who remained in Japan after US repatriation flights, by type of criteria met for PHTR removal. Horizontal line within the box is the median; bottom line of box is first quartile (25%), top line of box is third quartile (75%). Whiskers represent the minimum (bottom) and maximum (top) number of days. Dots represent outliers. PHTR, public health travel restrictions; SARS-COV-2, severe acute respiratory syndrome coronavirus 2.

Of the 69 patients with COVID-19, 40 recovered patients were able to meet the original criteria for PHTR removal, and 29 met the modified criteria. The median time from PHTR placement to removal was 17 days (range 7–57 days) (Appendix Figure 2). The median time from notification of a positive test to PHTR removal was 25 days (range 12–62 days) (Figure 4). Multiple persons reported persistent positive test results after symptom resolution; complete information was not available for the full cohort. CDC removed all PHTR by April 15.

## Discussion

The Diamond Princess COVID-19 outbreak represents a unique event in public health history: the quarantine of thousands of persons aboard a cruise ship for a newly identified disease at a time when most countries had few imported cases but the epidemic rapidly became a pandemic. Lessons learned from the experience of implementing PHTR on an unprecedented scale can inform the future use of PHTR in outbreaks with pandemic potential.

The first critical lesson is that during a novel disease epidemic, the lack of information about the disease challenges the general practice that ethical use of PHTR requires evidence-based decisions regarding when persons may be contagious or at risk of becoming contagious (*6*). CDC routinely uses PHTR for diseases such as infectious tuberculosis or measles, in which case definitions and criteria for determining noninfectiousness are well established (*3*). During this incident, little was known about SARS-CoV-2 transmission; transmission dynamics in Wuhan, China, and during the severe acute respiratory syndrome coronavirus (2003) and Middle Eastern respiratory syndrome coronavirus (since 2012) epidemics were used in establishing criteria for PHTR removal.

As the COVID-19 epidemic unfolded, we observed notable challenges in identifying persons who might transmit SARS-CoV-2 during travel or contribute to cross-border spread of disease (*22*). Limited information was available to determine whether potentially exposed persons who remained asymptomatic were no longer at risk of becoming infected or when infected persons were no longer infectious because the optimal monitoring period had not yet been determined and it was possible to persistently test positive (*23*). Particular challenges included differences in strategies for discontinuing isolation between the United States and Japan, differences in SARS-CoV-2 testing procedures between hospitals and laboratories in Japan and obtaining medical records and English translations (*24*). These challenges highlight the importance of multinational coordination at all levels and sectors. CDC used the best available data to modify criteria as needed through the event, ensuring that PHTR removal criteria were both evidence based and feasible in the international setting. The modified criteria simplified the PHTR removal process for many infected persons, and the time to remove PHTR was only 5 days longer for those meeting modified criteria (Figure 4). In this experience, clear and consistently applied criteria for removal of PHTR were essential, as was the flexibility to overcome the systemic challenges to meeting those criteria.

Second, there are logistical challenges in applying and monitoring travel restrictions on this scale. US government personnel had to identify each person for whom travel restrictions were warranted, verify their identities against federal databases, add them individually to the DNB and PHLO, document that criteria for removal of PHTR were met, and ensure timely removal of PHTR once the criteria were met. Because this situation took place on a cruise ship that kept detailed passenger and crew manifests, it was relatively straightforward to identify, notify, and obtain contact information for the persons at risk. If exposure had occurred in a less well-defined situation, identification would have been particularly challenging. However, the complex process to add >100 persons to the DNB and PHLO in the compressed timeframe of 24–48 hours required substantial federal resources.

Typically, documenting criteria for PHTR removal is completed by US jurisdictions or foreign public health authorities (*1*–*3*). For this event, CDC independently created a mechanism to document that exposed persons remained asymptomatic at the end of the 14-day period through an active monitoring process that continued past the end of Japan’s mandated quarantine period. Despite rapid implementation, CDC’s monitoring process worked well: most travelers had access to email either directly through their personal smartphones or phones provided by the cruise line or through family members in the United States who could communicate with them, and they responded to daily CDC information requests. The USEMB Japan could track the status of hospitalized patients, although this process required substantial resources for translation. Monitored persons and the involved agencies coordinated a large volume of communication both in managing emails with active monitoring information from Diamond Princess travelers and responding to individual travelers’ queries about their situations. However, in situations in which this level of coordination is unlikely or persons are difficult to contact, active monitoring for the purposes of PHTR may be challenging to accomplish (*25*,*26*). Before considering use of PHTR, especially on a large scale, implementors should evaluate what resources or capacity are available for identifying persons to be placed on PHTR, personnel required across all relevant agencies and countries to monitor travelers’ status and document when they are eligible for removal of PHTR, diagnostic testing capacity, and communications channels.

Third, it is critical that travel restrictions do not create undue risk to affected persons. Persons with positive tests or who became ill could access high-quality medical care in Japan; ethics considerations could be different for persons seeking medical care in areas with inadequate medical infrastructure. In such situations, charter travel or medical evacuation may be options for US citizens and residents to return safely to the United States (*1*), but this option is challenging on a large scale and may have been especially difficult in this situation, in which some of the patients remaining in Japan had complex medical needs. In this situation, the cruise line covered most travel expenses incurred by restricted travelers; CDC can assist travelers whose travel is restricted or delayed for public health reasons by requesting airlines to waive rebooking fees.

Limitations of the analysis were incomplete information, including hospital locations and clinical severity, and inability to obtain the specimen collection date. We approximated date of positive test result by the date of notification.

As with all decisions, overall costs and benefits should be considered when determining whether to use PHTR. Protection of the public, individual civil liberties, and increasing risk for disease transmission are all considerations that can inform use of PHTR (*7*). Costs and benefits may vary as outbreaks or pandemics progress; the benefit was evident in this event when the United States had only 15 cases. By mid-March 2020, the United States had reported over 6,000 confirmed cases of COVID-19. Most states implemented community mitigation measures such as social distancing requirements and cancellation of mass gatherings, especially once the World Health Organization declared COVID-19 a pandemic on March 11 (*27*,*28*).

PHTR implementation may have prevented transmission via air travel among Diamond Princess travelers under active monitoring and never tested. Transmission on the cruise ship was possible for many days before the vessel quarantine began; it is likely there were many asymptomatic but SARS-CoV-2–positive persons aboard (*6*). As the domestic outbreak grew, CDC shifted to different containment strategies for cruise ships on which COVID-19 cases occurred, including requirements for noncommercial travel after disembarkation and a No Sail Order for all cruise ships operating in US waters (*29**–**31*).

CDC continues to use individual-level PHTR for persons with known or suspected COVID-19 or high-risk exposures and advises persons who are symptomatic, test positive for SARS-CoV-2, or have been exposed to someone with COVID-19 to delay travel until no longer infectious or at risk of becoming infectious. CDC shares information and considerations for use of PHTR with health authorities of other countries but recognizes that many countries may not have the resources to manage similar systems. Lessons learned from the Diamond Princess outbreak are essential in planning future PHTR use during this pandemic or future outbreaks of novel diseases.

Appendix Additional information about US public health travel restrictions placed on passengers and crew during the COVID-19 outbreak on the Diamond Princess cruise ship, Japan, February–April 2020.
