# Supplementary material for: Use of US Public Health Travel Restrictions during COVID-19 Outbreak on Diamond Princess Ship, Japan, February–April 2020
Source: Emerg Infect Dis. 2021 Mar;27(3):710–8. doi: 10.3201/eid2703.203820 (PMC7920645; doi:10.3201/eid2703.203820)
Supplement: Appendix — Additional information about US public health travel restrictions placed on passengers and crew during the COVID-19 outbreak on the Diamond Princess cruise ship, Japan, February–April 2020. [file 20-3820-Techapp-s1.pdf]

Article DOI: https://doi.org/10.3203/eid2703.203820

Use of US Public Health Travel Restrictions during COVID-19 Outbreak on Diamond Princess Ship, Japan, February–April 2020

Appendix

A

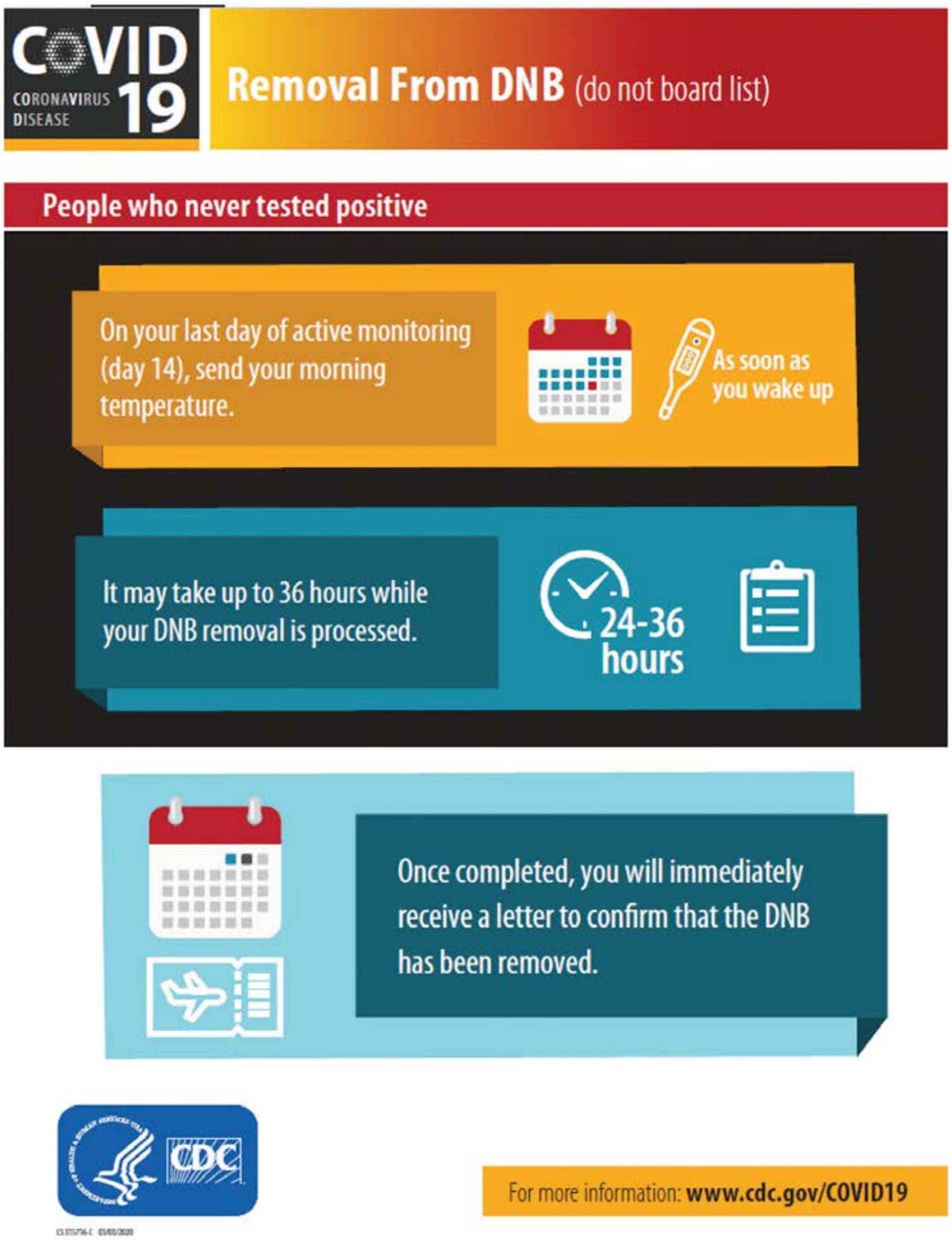

B

COVID 19 CORONAVIRUS DISEASE

Removal From DNB (do not board list)

People who previously tested positive (American Testing Criteria)*

After you report your second set of negative test results to CDC, it may take up to 36 hours while your DNB removal is processed.

24-36 hours

Once completed, you will immediately receive a letter to confirm that the DNB has been removed.

*1. Resolution of fever, without use of fever-reducing medication, 2. Improvement of illness symptoms, and 3. Two negative sets of test results from both a nasal swab and a throat swab taken at least 24 hours apart.

For more information: **www.cdc.gov/COVID19**

C

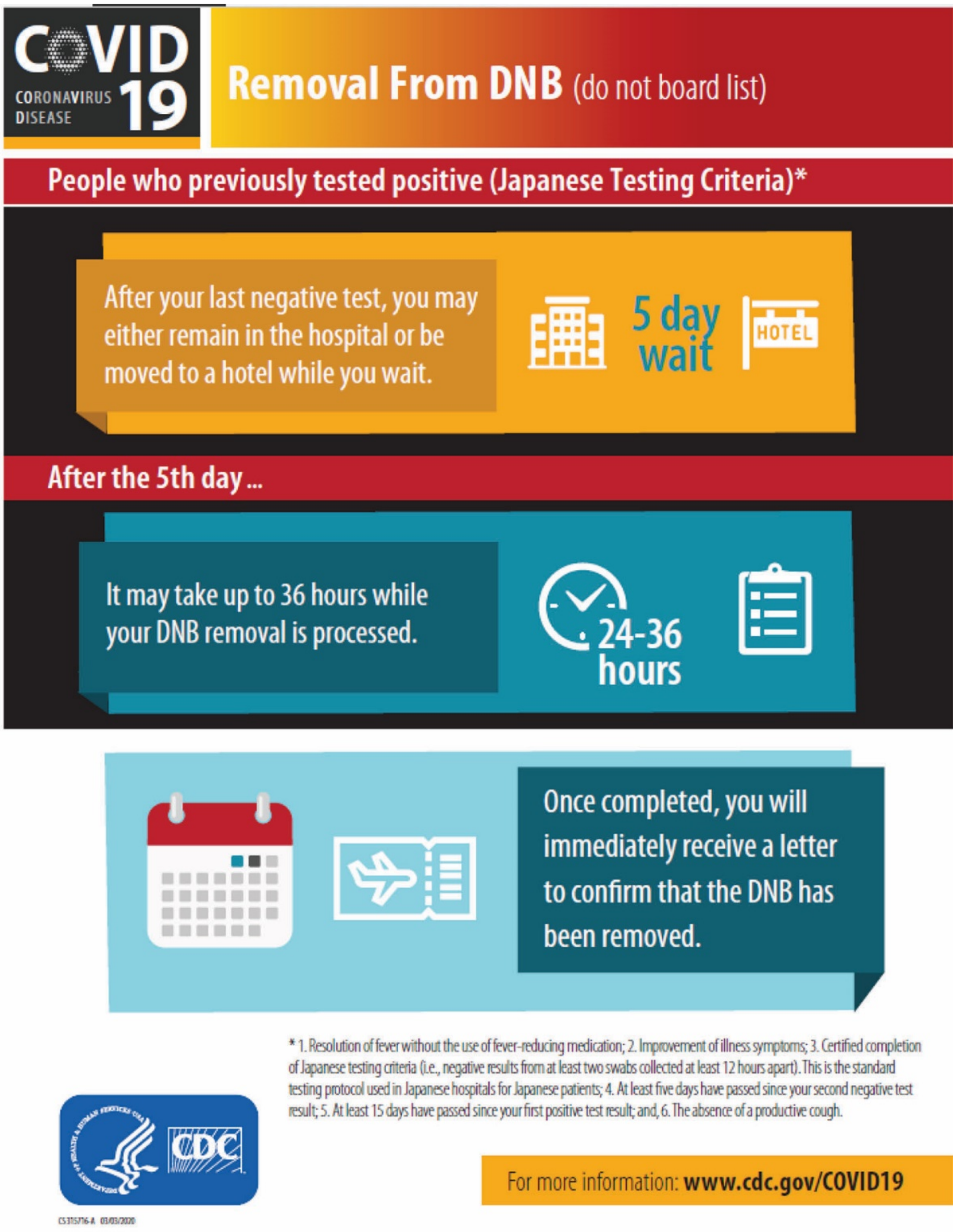

Appendix Figure 1. Guidelines for removal from Do Not Board list for US citizens and residents from the Diamond Princess cruise ship during the coronavirus disease (COVID-19) outbreak, Japan, February–April 2020. A) Guidelines for persons who never tested positive for COVID-19. B) Guidelines for persons who tested positive by US criteria. C) Guidelines for persons who tested positive by Japanese criteria.

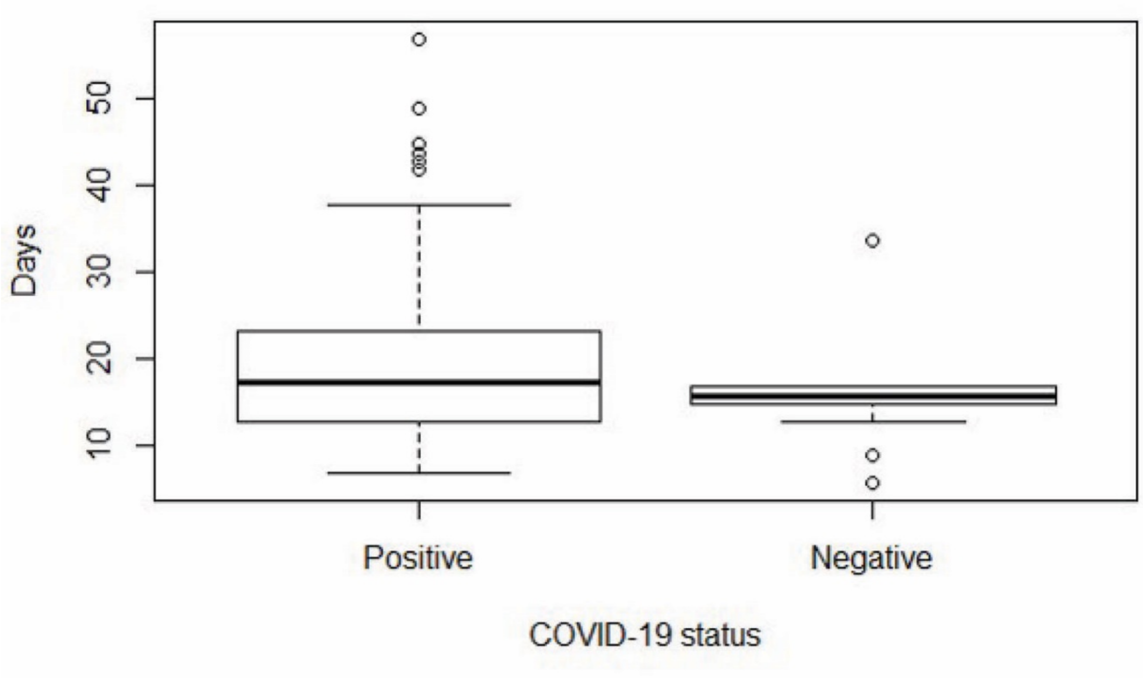

Appendix Figure 2. Days between placement and removal of public health travel restrictions for passengers and crew on board the Diamond Princess cruise ship during the coronavirus disease (COVID-19) outbreak, February 2020. Results are shown for persons who remained in Japan after US repatriation flights, by COVID-19 test result.
